# Rethinking Social Relationships: The Differential Investment of Resources Model of Social Development

**DOI:** 10.1093/geroni/igaa057.1309

**Published:** 2020-12-16

**Authors:** Katherine Fiori, Tim Windsor, Oliver Huxhold

**Affiliations:** 1 Adelphi University, Garden City, New York, United States; 2 Flinders University, Adelaide, South Australia, Australia; 3 German Centre of Gerontology, Berlin, Berlin, Germany

## Abstract

The empirical evidence concerned with the centrality of social relations to individual functioning across adulthood continues to accumulate. Theoretical development about age-related changes in social relationships, however, has lagged behind. In particular, existing theories either do not account for the influence of the developmental context, or are difficult to examine empirically because they do not posit specific testable mechanisms. We present a new conceptual model that we believe effectively incorporates much of the existing empirical work. The Differential Investment of Resources (DIRe) model has five distinct features. First, the model distinguishes between different types of ‘social ties’ by defining two crucial dimensions - closeness and kinship. Second, the investment of time and energy is defined as the core mechanism that explains the formation and maintenance of social ties. Third, individual characteristics, categorized as capacities, motivations, and skills, determine the amount, direction, and efficacy of the time and energy invested. Fourth, the model incorporates the developmental context: (a) in its effect on the social opportunity structure; (b) in its effect on time and energy; and (c) in its effect on the individual. Additionally, the social opportunity structure itself is determined by the individual’s existing social network ties (i.e., social capital). Finally, the model describes how different types of ties, in turn, affect individual characteristics via social functions (social exchanges, social evaluations, and social influences). The proposed model will not only stimulate a healthy new debate in the field, but will also provide a theoretical basis for future research and hypothesis-testing.

